# Schisandrin B Prevents Hind Limb from Ischemia-Reperfusion-Induced Oxidative Stress and Inflammation via MAPK/NF-*κ*B Pathways in Rats

**DOI:** 10.1155/2017/4237973

**Published:** 2017-06-19

**Authors:** Ning Zhu, Changhong Cai, Aiming Zhou, Xuyong Zhao, Yijia Xiang, Chunlai Zeng

**Affiliations:** ^1^Department of Cardiology, The Fifth Affiliated Hospital of Wenzhou Medical University, Lishui Central Hospital, Zhejiang Province 323000, China; ^2^Department of Cardiology, The Third Clinical College of Wenzhou Medical University, Wenzhou People's Hospital, Wenzhou, Zhejiang Province 325000, China

## Abstract

Schisandrin B (ScB), isolated from* Schisandra chinensis* (*S. chinensis*), is a traditional Chinese medicine with proven cardioprotective and neuroprotective effects. However, it is unclear whether ScB also has beneficial effects on rat hind limb ischemia/reperfusion (I/R) injury model. In this study, ScB (20 mg/kg, 40 mg/kg, and 80 mg/kg) was administered via oral gavage once daily for 5 days before the surgery. After 6 h ischemia and 24 h reperfusion of left hind limb, ScB reduced I/R induced histological changes and edema. ScB also suppressed the oxidative stress through decreasing MDA level and increasing SOD activity. Moreover, above changes were associated with downregulated TNF-*α* mRNA expression and reduced level of IL-1β in plasma. Meanwhile, ScB treatment downregulated activation of p38MAPK, ERK1/2, and NF-*κ*B in ischemic skeletal muscle. These results demonstrate that ScB treatment could prevent hind limb I/R skeletal muscle injury possibly by attenuating oxidative stress and inflammation via p38MAPK, ERK1/2, and NF-*κ*B pathways.

## 1. Introduction

The rat hind limb ischemia-reperfusion (I/R) injury and extremity vascular injury model resembles human diseases including extremity vascular injury [1], peripheral artery disease [2], and tourniquet application [3], which might sometimes lead to multiple organ failure and death.

Although the mechanisms of hind limb I/R injury are complicated, accumulating evidence has suggested that reactive oxygen species (ROS) and inflammation play a crucial role in the pathogenesis of hind limb I/R injury. I/R injury can promote the formation and the release of various inflammatory cytokines, such as tumor necrosis factor alpha (TNF-*α*) and interleukin 1 beta (IL-1*β*) [4], stimulate the process of the lipid peroxidation of biological membranes, and induce toxic metabolites formation such as malondialdehyde (MDA) [5]. Various defense mechanisms will be induced by ROS-induced injury [6] and superoxide dismutase (SOD) is the best known antioxidant enzyme capable of scavenging ROS [7]. P38 MAPK can be activated under various environmental stress and inflammation [8, 9]. It has been reported that activation of p38 and ERK1/2 was involved in renal ischemic reperfusion injury [10]. Furthermore, blockade of P38 *α* and *β* may protect lung from acute injury in II/R by reducing the expression of IL-1*β* [11]. Moreover, 6-gingerol exerts protective effects against I/R induced intestinal mucosa injury via inhibiting the formation of ROS and activation of p38 MAPK and NF-*κ*B [12]. It was also reported that gypenoside protects cardiomyocytes against I/R injury through the inhibition of MAPK pathways and NF-*κ*B p65 translocation into nuclei [13].

*Schisandra chinensis* (*S. chinensis*) is a traditional Chinese herb and possesses diverse biological activities [14]. Schisandrin B (ScB), extracted from the fruit of* S. chinensis*, is the most important active dibenzocyclooctadiene derivative, commonly used for the treatment of hepatitis in China [15]. ScB has been shown to reduce oxidative stress and inflammation in doxorubicin induced cardiac dysfunction model through MAPK/p53 signaling [16] in mice. ScB treatment was also shown capable of enhancing cellular glutathione level and protecting against oxidant stress by MAPKs pathway [17] and reducing carbon tetrachloride-induced liver damage [18]. Furthermore, ScB treatment (2 mmol/kg p.o.) ameliorated the Imject Alum-induced peritonitis [19]. The beneficial effects of ScB against I/R injury have been proposed in the heart [20] and brain [21]; however, the underlying pharmacologic effect of ScB on hind limb I/R injury remains elusive now.

The aim of present study is to investigate if ScB treatment could ameliorate oxidative stress and inflammation through modulation of p38MAPK and ERK1/2 in the rat model of hind limb I/R.

## 2. Materials and Methods

### 2.1. Chemicals and Reagents

ScB was purchased from the National Institute for the Control of Pharmaceutical and Biological Products (Beijing, China, purity > 96%). The primary antibodies p-p38, p38, p-ERK1/2, ERK1/2, NF-*κ*B p65, and horseradish peroxidase-conjugated anti-rabbit secondary antibodies were from Cell Signaling Technology (Danvers, MA).

### 2.2. Experimental Groups and ScB Treatment

SD rats (250–300 g) used were purchased from Experimental Animal Center of Zhejiang Province. All animal studies described in this work have been approved by the Wenzhou Medical University Animal Policy and Welfare Committee. All experiments involving rats were carried out according to the National Institutes of Health (Bethesda, MD, USA) guidelines. The animals were housed at 22 ± 2°C under a 12-h light/12-h dark cycle with free access to food and water and were randomly divided into three groups as follows: (1) control group were given 5% Tween 20, (2) I/R injury group were given 5% Tween 20, (3) ScB treatment group were given ScB at 20 mg/kg body weight, (4) ScB treatment group were given ScB at 40 mg/kg body weight, and (5) ScB treatment group were given ScB at 80 mg/kg body weight. ScB was dissolved in 5% Tween 20 and given daily by oral gavage 5 days before the surgery.

### 2.3. I/R Model

After the animals were anesthetized with sodium pentobarbital (30 mg/kg, IP), the left hind limb was prepared for surgery. Rat model of hind limb ischemia-reperfusion injury was conducted as previous report [22], and briefly hind limb I/R injury was induced by 6 h of femoral artery occlusion, followed by 24 h of reperfusion.

### 2.4. Morphometric Analysis

Skeletal muscles were collected from the experimental group rats and immersed in 4% paraformaldehyde overnight and then embedded in paraffin. Paraffin blocks were sliced into sections of 4 *μ*m in thickness and stained with hematoxylin and eosin (H&E). Then each section was imaged by a microscopy (Nikon, Japan). The degree of histologic damage was evaluated morphologically and quantitatively. Inflammatory cell infiltration in the airspace or vessel wall, alveolar congestion, hemorrhage, alveolar wall thickness, and hyaline membrane formation were the parameters used to score the evaluation. A score of 0 represented no damage; l represented mild damage; 2 represented moderate damage; 3 represented severe damage; and 4 represented very severe histologic changes, and total score of damage was quantified by the sum of each of the parameters [23].

### 2.5. Assessment of Edema Formation

The severity of skeletal muscle edema was assessed by the wet to dry ratio. The muscle samples were taken and immediately weighed to obtain the wet weight, and dry weight was measured after the tissues dried for 72 h at 60°C.

### 2.6. MDA and SOD Measurements

Tissue samples were homogenized in PBS (pH = 7.4) to make a 10% homogenate. The homogenates were centrifuged at 5000 rpm for 10 min and the supernatants were collected. In tissue samples, the levels of MDA and SOD activity were detected as previously described [24].

### 2.7. ELISA Assessment of IL-1*β* and TNF-*α*

Blood drawn from animals was centrifuged at 3000 rpm for 10 min and stored at −80°C until analysis. ELISAs for IL-1*β* and TNF-*α* (Bio-Swamp, Shanghai, China) were carried out on the serum samples. Then samples were assayed immediately following the procedure recommended by the manufacturer.

### 2.8. Quantitative Real-Time RT-PCR

Total RNA was isolated from the serum samples. Reverse transcription and quantitative PCR (RT-qPCR) was carried out using MMLV Platinum RT-qPCR kit (Life Technologies). Real-time qPCR was performed using the Eppendorf Real plex 4 instrument (Eppendorf, Hamburg, Germany). Primers for genes including TNF-*α*, IL-1, and GAPDH were obtained from Life Technologies. The relative amount of each gene was normalized to the amount of GAPDH. The primer sequences used are shown in Table 1.

### 2.9. Western Blot Analysis

Skeletal muscle tissues were harvested, stored in lysis buffer with protease inhibitors, and phosphatase inhibitor, and frozen in liquid nitrogen immediately until homogenization. Total protein samples were separated by 10% SDS–PAGE gel electrophoresis and then transferred to nitrocellulose membranes (Bio-Rad, Hercules, CA). The membranes were blocked with 5% bovine serum albumin in 10 mM Tris–HCl containing 150 mM NaCl and 0.5% Tween 20 (TBST) and then incubated with primary antibodies against p-p38MAPK, p38MAPK, p-ERK1/2, ERK1/2, p-JNK, JNK, and NF-*κ*B p65, respectively. After the incubation with second antibodies, immunostained bands were visualized by using ECL kit (BioRad, Hercules, CA).

### 2.10. Statistical Analysis

The results were expressed as mean ± SD. For comparison of multiple groups, one-way analysis of variance (ANOVA) was used with Bonferroni correction using GraphPad Prism version 5.0 for Windows (GraphPad Software, Inc., San Diego, CA). Differences with *P* < 0.05 were considered statistically significant.

## 3. Results

### 3.1. ScB Ameliorates Ischemic Histological Changes and Edema of Skeletal Muscle

**Figure 1:**
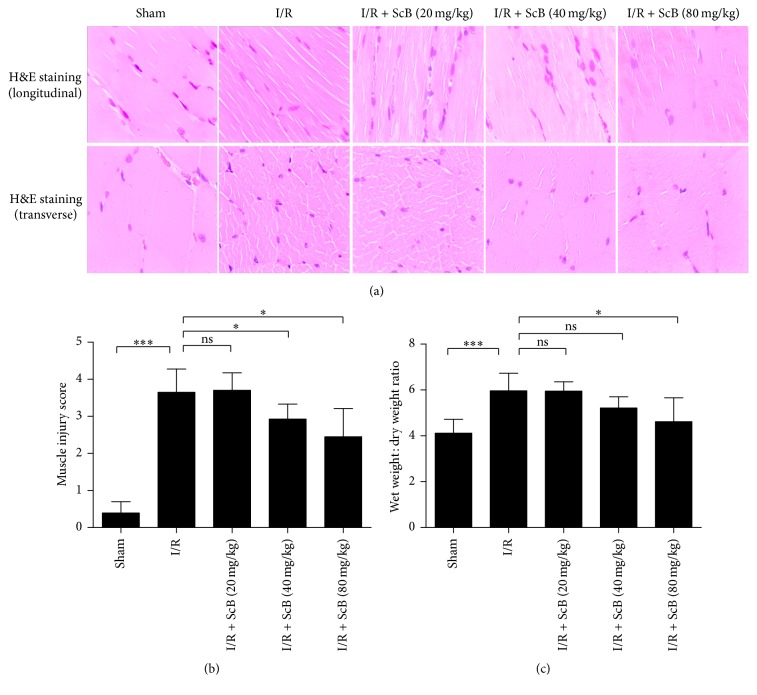
ScB ameliorated skeletal muscle damage and edema following left hind limb I/R. (a) Representative images from longitudinal and transverse H&E stained skeletal muscle tissues (400x). (b) Histopathological mean skeletal muscle injury scores determined from low-power (×20) microscopic view. (c) Edema presented as wet weight/dry weight ratio of left hind limb skeletal muscle. ^*∗∗∗*^*P* < 0.001 versus sham, ^*∗*^*P* < 0.05 versus I/R, *n* = 6 per group.

Hematoxylin and eosin (H&E) stained sections showed that I/R group displayed typical I/R induced structural abnormalities in longitudinal and transverse cross-sections (Figure 1(a)). ScB (80 mg/kg) treatment significantly attenuated pathological damage in left hind limb muscle. Both skeletal muscle injury score and skeletal muscle wet/dry ratio were significantly lower in ScB (80 mg/kg) group compared to I/R group (both *P* < 0.05, Figures 1(b) and 1(c)).

### 3.2. ScB Treatment Reduces MDA and Increases SOD Activity

**Figure 2:**
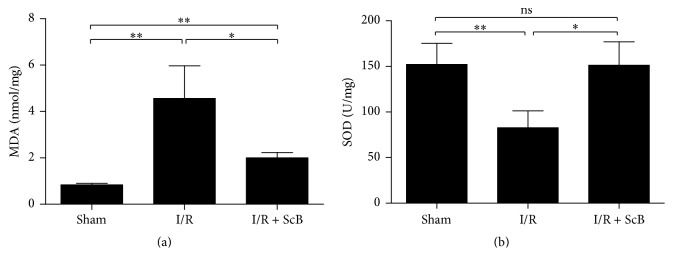
ScB reduced MDA content and enhanced SOD activity in the skeletal muscle following hind limb I/R. (a) MDA content and (b) SOD activity in the ischemic skeletal muscle after 24 h reperfusion. ^*∗∗*^*P* < 0.01 versus sham, ^*∗*^*P* < 0.05 versus I/R, *n* = 6 per group.

As shown in Figure 2(a), the tissue level of MDA was significantly increased in I/R group compared to control group (*P* < 0.05), which could be significantly reduced by ScB (80 mg/kg) treatment (*P* < 0.05 versus I/R).

In contrast, SOD activity was significantly reduced in I/R group compared to control group (*P* < 0.05), which could be significantly increased by ScB (80 mg/kg) treatment (*P* < 0.05 versus I/R, Figure 2(b)).

### 3.3. ScB Treatment Attenuates Plasma Inflammatory Cytokines

**Figure 3:**
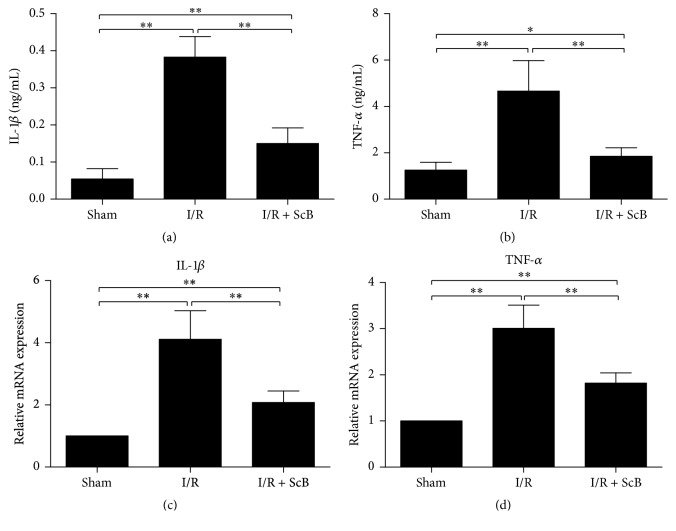
ScB attenuated plasma TNF-*α* and IL-1*β* levels (a, b) and mRNA expression of TNF-*α* and IL-1*β* (c, d) in plasma following hind limb I/R expression and mRNA levels of TNF-*α*. ^*∗∗*^*P* < 0.01 versus sham, ^*∗∗*^*P* < 0.01 versus I/R, *n* = 6 per group, and ^*∗*^*P* < 0.05 versus sham.

Plasma levels and mRNA expression levels of TNF-*α* and IL-1*β* were determined by ELISA and real-time PCR (Figure 3). Compared with the control group, plasma levels and the mRNA expression levels of TNF-*α* and IL-1*β* were both significantly upregulated in I/R group compared to control group (*P* < 0.05), which could be significantly downregulated by ScB (80 mg/kg) treatment (*P* < 0.05).

### 3.4. ScB Treatment Suppresses Activation of p38MAPK, ERK1/2, JNK, and NF-*κ*B p65

**Figure 4:**
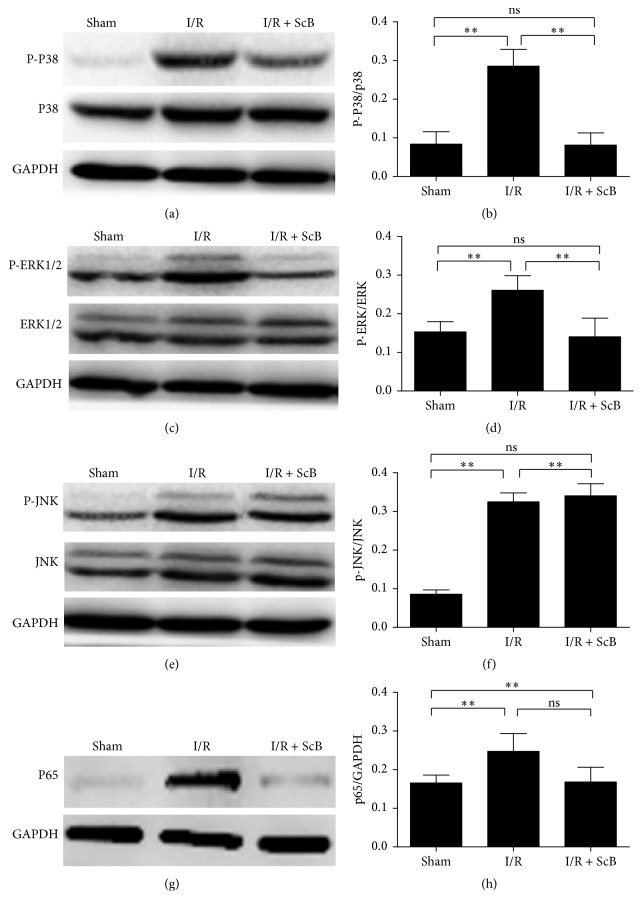
ScB reduced activation of p38, ERK1/2, JNK, and NF-*κ*B in the skeletal muscle following hind limb I/R. (a, b) Representative immunoblots and densitometric analysis of p-p38MAPK, total p38, and GAPDH in the ischemic skeletal muscle after 24 h reperfusion. (c, d) Representative immunoblots and densitometric analysis of p-ERK1/2, total ERK1/2, and GAPDH in the ischemic skeletal muscle after 24 h reperfusion. (e, f) Representative immunoblots and densitometric analysis of p-JNK, total JNK, and GAPDH in the ischemic skeletal muscle after 24 h reperfusion. (g, h) Representative immunoblots and densitometric analysis of NF-*κ*B p65 and GAPDH in the ischemic skeletal muscle after 24 h reperfusion. ^*∗∗*^*P* < 0.01 versus sham, ^*∗∗*^*P* < 0.01 versus I/R, *n* = 6 per group.

To further investigate the molecular mechanisms underlying the anti-inflammatory and anti-ROS effects of ScB (80 mg/kg), Western blot analysis was performed to determine the potential role of p38MAPK, ERK1/2, JNK, and NF-*κ*B p65 activation in left hind limb tissue samples of various groups. As shown in Figure 4, I/R injury elicited an apparent upregulation of phosphorylated p38MAPK, ERK1/2, and JNK as well as the expression of NF-*κ*B p65 (all *P* < 0.01) in ischemic skeletal muscle samples, while ScB administration significantly reduced I/R induced activation of p38MAPK, ERK1/2, and NF-*κ*B p65 (all *P* < 0.01) but not JNK (*P* > 0.05) in left hind limb samples, indicating that the protective role of ScB in this skeletal I/R injury model might be partly mediated through inhibiting p38MAPK, ERK1/2, and NF-*κ*B p65 pathways.

## 4. Discussion

Clinical skeletal muscle I/R injury could be elicited by multiple pathological etiologies, which can cause significant injury with serious remote organ dysfunction [25]. There are some clinical strategies aiming to attenuate the detrimental effects of hind limb I/R injury; however, the efficacy achieved is far from satisfaction [26]. In the present study, the effects of ScB on femoral arterial clamping-induced skeletal muscle I/R injury were investigated. We found that ScB treatment can significantly attenuate the hind limb I/R injury in this model. Histologic finding revealed significantly less histopathologic changes in ScB treated animals. Moreover, we found that ScB led to significant reduction of edema in ischemic skeletal muscle. ScB at a dosage of 80 mg/kg exerted the strongest effects on hind limb I/R injury. Hence, we wanted to explore the mechanisms of the effects of ScB (80 mg/kg) on skeletal muscle damage. Our results demonstrated that ScB treatment reduced MDA level and increased SOD activity and attenuated plasma inflammatory cytokines expression and ScB administration also significantly reduced I/R induced activation of p38MAPK, ERK1/2, and NF-*κ*B p65 but not JNK, indicating that the protective role of ScB in this skeletal I/R injury model might be partly mediated through inhibiting p38MAPK, ERK1/2, and NF-*κ*B p65 pathways. To the best of our knowledge, this is the first report demonstrating the beneficial effects and the potential mechanism of ScB in this rat model of hind limb I/R injury.

The role of oxidative stress in the setting of hind limb I/R injury is well demonstrated in previous studies [27–29]. Hence, we investigated the effects of ScB on the level of MDA and SOD activity. Our data showed that the level of MDA was increased in I/R group compared with control group, while SOD activity was reduced. Administration of ScB could decrease the level of MDA and increase SOD activity, our results thus suggesting that ScB could protect against ROS-induced injury in this model.

Proinflammatory mediators, such as TNF-*α* and IL-1*β*, rapidly released from injured tissue in the initial phase of hind limb I/R are known to play a crucial role in hind limb I/R injury [30]. These cytokines could recruit inflammatory cells and regulate the permeability of blood and lymphatic vessels and lead to endothelial dysfunction [31, 32]. This process is one of the key features of the immunological reaction to hind limb I/R injury. Previous study showed that reduction of these cytokines could effectively attenuate hind limb I/R injury [33]. In line with above findings, present study also demonstrated that treatment with ScB was capable of diminishing I/R induced TNF-*α* and IL-1*β* increases. This result suggested that ScB reduced skeletal muscle damage in hind limb I/R injury possibly by reducing the I/R induced increase of TNF-*α* and IL-1*β*.

Mitogen-activated protein kinase includes extracellular signal-regulated kinases (ERK1 and ERK2), c-jun-N-terminal kinase (JNK1 and JNK2), and p38 MAPK. P38MAPK and ERK1/2 may regulate oxidative stress and inflammatory responses [34, 35]. It is known that many I/R injury models elicited the activation of p38MAPK and ERK1/2 pathways [36–38]. However, the role of p38MAPK and ERK1/2 signaling in hind limb I/R injury is not fully understood now. ScB has been shown to efficiently scavenge oxygen free radicals, inhibit inflammation, and protect DNA from oxidative damage [16]. Accordingly, ScB was capable of reducing myocardial I/R injury and cerebral I/R injury (ref). Furthermore, following production of ROS and activation of p38MAPK and ERK1/2, NF-*κ*B could translocate to the nucleus, where they upregulate gene expression of proinflammatory mediators such as IL-1 and TNF-*α* [39, 40]. Hence, we examined whether ScB could regulate the expression of p-p38MAPK, p-ERK1/2, and NF-*κ*B P65 in the skeletal muscle after I/R injury. As expected, activated p38, ERK1/2, and NF-*κ*B P65 could be partially downregulated by ScB, which implied that modulation of p38MAPK, ERK1/2, and NF-*κ*B signaling pathway might be one of the working mechanisms underlying the protective effects of ScB in this model.

In conclusion, our data demonstrate that anti-inflammatory and antioxidative effects of ScB might be mediated by inhibiting the phosphorylation of p38MAPK and ERK1/2 and activation of NF-*κ*B in this model. ScB may be a promising drug for attenuating tissue damage in hind limb I/R injury in a clinical setting.

The limitation of our study is further research of the role of p38MAPK, ERK1/2, and NF-*κ*B in hind limb I/R injury, antioxidative stress, and inflammation.

## Figures and Tables

**Table 1:** List of gene-specific primer sequences.

Gene	Sequence
IL-1*β*	Forward: ATCTCACAGCAGCATCTC Reverse: TAGCAGGTCGTCATCATC
TNF-*α*	Forward: TGGCGTGTTCATCCGTTC Reverse: CTACTTCAGCGTCTCGTGTG
GAPDH	Forward: GTCGGTGTGAACGGATTTG Reverse: TCCCATTCTCAGCCTTGAC

## References

[1] Percival T. J., Rasmussen T. E. (2012). Reperfusion strategies in the management of extremity vascular injury with ischaemia. *British Journal of Surgery*.

[2] Hiatt W. R., Armstrong E. J., Larson C. J., Brass E. P. (2015). Pathogenesis of the limb manifestations and exercise limitations in peripheral artery disease. *Circulation Research*.

[3] Estebe J.-P., Davies J. M., Richebe P. (2011). The pneumatic tourniquet: Mechanical, ischaemia-reperfusion and systemic effects. *European Journal of Anaesthesiology*.

[4] Seekamp M. D., Ward P. A. (1993). Ischemia-reperfusion injury. *Agents and Actions. Supplements*.

[5] Concannon M. J., Kester C. G., Welsh C. F., Puckett C. L. (1992). Patterns of free-radical production after tourniquet ischemia: implications for the hand surgeon. *Plastic and Reconstructive Surgery*.

[6] McCord J. M. (2000). The evolution of free radicals and oxidative stress. *The American Journal of Medicine*.

[7] Bulkley G. B. (1993). Free radicals and other reactive oxygen metabolites: clinical relevance and the therapeutic efficacy of antioxidant therapy. *Surgery*.

[8] Guo L., Wang L., Li H. (2016). Down regulation of GALNT3 contributes to endothelial cell injury via activation of p38 MAPK signaling pathway. *Atherosclerosis*.

[9] Jacquet S., Zarrinpashneh E., Chavey A. (2007). The relationship between p38 mitogen-activated protein kinase and AMP-activated protein kinase during myocardial ischemia. *Cardiovascular Research*.

[10] Zhang J., Xia J., Zhang Y. (2016). HMGB1-TLR4 signaling participates in renal ischemia reperfusion injury and could be attenuated by dexamethasone-mediated inhibition of the ERK/NF-*κ*B pathway. *American Journal of Translational Research*.

[11] Zheng D. Y., Zhou M., Jin J. (2016). Inhibition of P38 MAPK downregulates the expression of IL-1*β* to protect lung from acute injury in intestinal ischemia reperfusion rats. *Mediators of Inflammation*.

[12] Li Y., Xu B., Xu M. (2017). 6-Gingerol protects intestinal barrier from ischemia/reperfusion-induced damage via inhibition of p38 MAPK to NF-*κ*B signalling. *Pharmacological Research*.

[13] Yu H., Shi L., Qi G., Zhao S., Gao Y., Li Y. (2016). Gypenoside protects cardiomyocytes against ischemia-reperfusion injury via the inhibition of mitogen-activated protein kinase mediated nuclear factor kappa B pathway in vitro and in vivo. *Frontiers in Pharmacology*.

[14] Panossian A., Wikman G. (2008). Pharmacology of Schisandra chinensis Bail.: an overview of Russian research and uses in medicine. *Journal of Ethnopharmacology*.

[15] Liu G. t. (1989). Pharmacological actions and clinical use of fructus schizandrae. *Chinese Medical Journal*.

[16] Thandavarayan R. A., Giridharan V. V., Arumugam S. (2015). Schisandrin B prevents doxorubicin induced cardiac dysfunction by modulation of DNA damage, oxidative stress and inflammation through inhibition of mapk/p53 signaling. *PLoS One*.

[17] Leong P. K., Chiu P. Y., Chen N., Leung H., Ko K. M. (2011). Schisandrin B elicits a glutathione antioxidant response and protects against apoptosis via the redox-sensitive ERK/Nrf2 pathway in AML12 hepatocytes. *Free Radical Research*.

[18] Chiu P. Y., Mak D. H., Poon M. K. T., Ko K. M. (2005). Role of cytochrome P-450 in schisandrin B-induced antioxidant and heat shock responses in mouse liver. *Life Sciences*.

[19] Leong P. K., Ko K. M. (2015). Schisandrin B induces an Nrf2-mediated thioredoxin expression and suppresses the activation of inflammasome in vitro and in vivo. *BioFactors*.

[20] Chen P., Pang S., Yang N. (2013). Beneficial effects of schisandrin B on the cardiac function in mice model of myocardial infarction. *PLoS One*.

[21] Lee T. H., Jung C. H., Lee D. H. (2012). Neuroprotective effects of Schisandrin B against transient focal cerebral ischemia in Sprague-Dawley rats. *Food and Chemical Toxicology*.

[22] Takhtfooladi H., Takhtfooladi M., Moayer F., Mobarakeh S. (2015). Melatonin attenuates lung injury in a hind limb ischemia-reperfusion rat model. *Revista Portuguesa de Pneumologia*.

[23] Zhu T., Wang D. X., Zhang W. (2013). Andrographolide protects against LPS-Induced Acute Lung Injury by Inactivation of NF-*κ*B. *PLoS One*.

[24] Kirisci M., Oktar G. L., Ozogul C. (2013). Effects of adrenomedullin and vascular endothelial growth factor on ischemia/reperfusion injury in skeletal muscle in rats. *Journal of Surgical Research*.

[25] Harris K., Walker P. M., Mickle D. A. (1986). Metabolic response of skeletal muscle to ischemia. *American Journal of Physiology*.

[26] Percival T. J., Rasmussen T. E. (2012). Reperfusion strategies in the management of extremity vascular injury with ischaemia. *British Journal of Surgery*.

[27] Wang X. T., Tian Y., Xu W. X. (2015). Protective effects of modeled superoxide dismutase coordination compound (MSODa) against ischemia/reperfusion injury in rat skeletal muscle. *Cellular Physiology and Biochemistry*.

[28] Takhtfooladi M. A., Asghari A., Takhtfooladi H. A., Shabani S. (2015). The protective role of curcumin on testicular tissue after hindlimb ischemia reperfusion in rats. *International Urology and Nephrology*.

[29] Takhtfooladi M. A., Jahanshahi A., Sotoudeh A., Daneshi M. H., Khansari M., Takhtfooladi H. A. (2013). The antioxidant role of n-acetylcysteine in testicular remote injury after skeletal muscle ischemia and reperfusion in rats. *Polish Journal of Pathology*.

[30] Zhao Y., Feng Q., Huang Z. (2014). Simvastatin inhibits inflammation in ischemia-reperfusion injury. *Inflammation*.

[31] Chu C., He W., Kuang Y., Ren K., Gou X. (2014). Celastrol protects kidney against ischemia-reperfusion-induced injury in rats. *Journal of Surgical Research*.

[32] He G. Z., Zhou K. G., Zhang R., Wang Y. K., Chen X. F. (2012). Impact of intestinal ischemia/reperfusion and lymph drainage on distant organs in rats. *World Journal of Gastroenterology*.

[33] Souza D. G., Cassali G. D., Poole S., Teixeira M. M. (2001). Effects of inhibition of PDE4 and TNF-*α* on local and remote injuries following ischaemia and reperfusion injury. *British Journal of Pharmacology*.

[34] Gupta J., Nebreda A. R. (2015). Roles of p38*α* mitogen-activated protein kinase in mouse models of inflammatory diseases and cancer. *FEBS Journal*.

[35] He W., Shi F., Zhou Z. W. (2015). A bioinformatic and mechanistic study elicits the antifibrotic effect of ursolic acid through the attenuation of oxidative stress with the involvement of ERK, PI3K/Akt, and p38 MAPK signaling pathways in human hepatic stellate cells and rat liver. *Drug Design, Development and Therapy*.

[36] Zhou L., Zhao D., An H., Zhang H., Jiang C., Yang B. (2015). Melatonin prevents lung injury induced by hepatic ischemia-reperfusion through anti-inflammatory and anti-apoptosis effects. *International Immunopharmacology*.

[37] Qi M., Zheng L., Qi Y. (2015). Dioscin attenuates renal ischemia/reperfusion injury by inhibiting the TLR4/MyD88 signaling pathway via up-regulation of HSP70. *Pharmacological Research*.

[38] Zhang S., Qi Y., Xu Y. (2013). Protective effect of flavonoid-rich extract from Rosa laevigata Michx on cerebral ischemia-reperfusion injury through suppression of apoptosis and inflammation. *Neurochemistry International*.

[39] Barnes P. J., Karin M. (1997). Nuclear factor-*κ*B—a pivotal transcription factor in chronic inflammatory diseases. *The New England Journal of Medicine*.

[40] Sun Z., Andersson R. (2002). NF-*κ*B activation and inhibition: a review. *Shock*.

